# How Terminology Affects Users’ Responses to System Failures

**DOI:** 10.1177/00187208231202572

**Published:** 2023-09-21

**Authors:** Cindy Candrian, Anne Scherer

**Affiliations:** Delta Labs AG, Zurich, Switzerland; URPP Social Networks, 30962 Faculty of Business, Economics and Informatics, University of Zurich, Switzerland

**Keywords:** trust in automation, system design, human-automation interaction, technology acceptance, expert systems

## Abstract

**Objective:**

The objective of our research is to advance the understanding of behavioral responses to a system’s error. By examining trust as a dynamic variable and drawing from attribution theory, we explain the underlying mechanism and suggest how terminology can be used to mitigate the so-called algorithm aversion. In this way, we show that the use of different terms may shape consumers’ perceptions and provide guidance on how these differences can be mitigated.

**Background:**

Previous research has interchangeably used various terms to refer to a system and results regarding trust in systems have been ambiguous.

**Methods:**

Across three studies, we examine the effect of different system terminology on consumer behavior following a system failure.

**Results:**

Our results show that terminology crucially affects user behavior. Describing a system as “AI” (i.e., self-learning and perceived as more complex) instead of as “algorithmic” (i.e., a less complex rule-based system) leads to more favorable behavioral responses by users when a system error occurs.

**Conclusion:**

We suggest that in cases when a system’s characteristics do not allow for it to be called ”AI,” users should be provided with an explanation of why the system’s error occurred, and task complexity should be pointed out. We highlight the importance of terminology, as this can unintentionally impact the robustness and replicability of research findings.

**Application:**

This research offers insights for industries utilizing AI and algorithmic systems, highlighting how strategic terminology use can shape user trust and response to errors, thereby enhancing system acceptance.

## INTRODUCTION

The adoption of algorithmic and Artificial Intelligence (AI) decision support systems has been accelerating, particularly when their predictions and forecasts yield superior accuracy compared to human-derived estimates. This is notably evident in tasks possessing well-defined objectives, such as stock market optimization (Zuckerman, 2019) and chess (Silver et al., 2017), where a substantial body of evidence supports the enhanced performance of intelligent systems. As a result, these systems are taking over various domains, including flight management systems (Parasuraman et al., 2000), GPS route planners (Hoff & Bashir, 2015), forecasts of employee performance (Highhouse, 2008), financial advice (Javelosa, 2017), sales processes (Davenport et al., 2020), and many more. These systems can be used to acquire and analyze information, make decisions, carry out actions, or even monitor other systems (Parasuraman et al., 2000), and reduce errors (Aron et al., 2011).

However, inevitably, systems occasionally fail. Although there is substantial evidence demonstrating that intelligent systems outperform human judgment, users often exhibit reluctance to depend on algorithmic solutions (Fildes & Goodwin, 2007) and generally exhibit hesitancy to restore trust in such systems after experiencing a service failure (Dietvorst et al., 2015). That is, seeing algorithmic systems err makes people less confident in them and less likely to choose the system again as compared to relying on a human, even though the latter’s performance is likely to be inferior. *Algorithm aversion* of this sort is costly at both the individual and societal levels, as it constitutes a barrier to the adoption of more effective, algorithm-based or AI approaches (Dietvorst et al., 2015). When humans and AI augment each other and work together, superior systems can be created (Jain et al., 2021). Hence, it is important to understand which factors affect how people lose and regain trust in systems after a failure.

While users’ algorithm aversion after a system error is well documented, we find several unaddressed areas within the prevailing literature, which we aim to address in this present research. First, trust dynamics in recurring interactions have been largely overlooked, leaving questions regarding the evolution of trust and users' behavioral responses to system errors. Second, the impact of system terminology on consumer responses to system failures remains underexplored. Third, the literature has not thoroughly investigated the underlying mechanism that drives trust reformation and satisfaction. Lastly, there is limited research on mitigating negative responses towards nonAI systems without resorting to “AI washing” (Bini, 2018; Moore, 2017). Although communicators often use terms such as algorithm or AI to convey similar concepts, the terminology chosen influences how people perceive and assess these systems. For instance, companies identified as operating in the “AI” domain secure 15%–50% more funding in their rounds compared to other technology startups (Vincent, 2019).

The present research aims to address these gaps. In particular, we will examine the underlying factors that influence users' responses to system failures, trust reformation, satisfaction, and reactions towards nonAI systems. Hereby, we will focus on the role of system terminology intrust reformation at varying levels of task difficulty and examine users' causal attributions of system failures as an underlying mechanism. By examining these factors, we aim to contribute to a deeper understanding of user responses to system failures and to inform practical applications for mitigating negative reactions.

Drawing from attribution theory (Weiner, 1985), we show how the perceived stability and complexity of a system affect trust reformation after a system failure. We show that the use of varying terminology and descriptions for algorithmic versus AI systems creates different expectations of system stability, which in turn affects the rebuilding of trust in a system after a failure. We argue that the effect of system type on trust is mediated by stability attributions, whereby AI systems are ascribed lower stability and rigidness than algorithmic systems, ascriptions which increase trust in the AI system. Additionally, we propose that this mediation is further influenced by the specific task environment, particularly task difficulty.

In a series of studies, we show that when seeing an algorithmic system err, users tend to ascribe higher stability to the system’s error, leading them to be more reluctant to trust the system again and resulting in an overall lower satisfaction with the system’s performance. This research thus makes an important contribution to the current understanding of algorithm aversion and its underlying mechanisms, ultimately contributing to the development of effective strategies for promoting user acceptance and trust in such systems.

## THEORETICAL DEVELOPMENT

### System Types

In this paper, we use the term “system” to denote any computational method that produces output for the user without human intervention. Systems can vary in complexity depending on the tasks they are designed to perform. The concept of “artificial intelligence” (AI) is prevalent in this field, but its definition remains contested. Prior research has identified definitions of AI that vary in inclusiveness (Bini, 2018; De Bruyn et al., 2020).

The broadest definition of AI is the intelligence exhibited by machines (Shieber, 2004). Brooks (1991) stated that AI aims to enable computers to perform tasks that, when executed by humans, are deemed intelligent. However, this broad definition can encompass anything from simple regression analysis to more complex AI applications. This ambiguity has led to companies claiming to offer AI-based products and services, a strategy known as “AI washing” (Moore, 2017), which is often met with skepticism by AI researchers. For instance, some elements of Salesforce’s “Einstein AI” rely on basic regression analyses.

The narrowest definition of AI proposes using the term “AI” exclusively for “artificial general intelligence” (AGI), defined as a machine’s ability to understand or learn any intellectual task a human can perform (Goertzel, 2014; Thórisson et al., 2015). Current AI applications focus on specific, limited tasks, such as chess, facial recognition, or ad click prediction. These applications, referred to as “weak AI” or “narrow AI,” do not learn beyond their programmed domains. In contrast, “strong AI” or “artificial general intelligence” involves machines that can “learn to learn” (Kurzweil, 2014).

De Bruyn et al. (2020) propose a balanced definition of AI as “machines that emulate human intelligence in tasks such as learning, planning, and problem-solving through higher-level, autonomous knowledge creation.” This definition offers several advantages, including the restriction of AI to algorithms that autonomously generate new constructs and knowledge structures. Anderson and Krathwohl’s (2021) revision of Bloom’s taxonomy of educational learning objectives places creation at the highest level of learning, which is key to distinguishing AI from traditional statistical techniques.

In this research, we adopt this balanced definition of AI and differentiate between *rule-based systems* and *self-learning systems*. Rule-based systems, or “algorithmic systems,” rely on predefined, expert-crafted heuristics to process input data and generate output. They provide consistency but cannot improve their forecasts as they rely on predefined rules described by algorithms. In contrast, AI systems, based on machine learning, learn patterns and strategies directly from data, adapting and improving performance based on input data and feedback. They encompass all self-learning systems that autonomously generate higher-order learning without human expert knowledge, in line with the prevalent understanding of AI (Kelnar & Kostadinov, 2019). In short, rule-based algorithms offer less flexibility, as they require manual intervention for updates, while self-learning AI adapts to new data or situations.

### Trust in Systems

Research on trust in systems has identified three key aspects impacting users’ trust: system characteristics, user characteristics, and the external environment (Hancock et al., 2011). Focusing on system characteristics, trust can be influenced by *features* of the system and *capabilities* of the system. One feature-based antecedent of trust is the level of automation, or the amount of control the automation is designed to exercise (Parasuraman et al., 2000). Prior research suggests that the relationship between level of control and trust is ambiguous (Cai et al., 2012; Merritt et al., 2013; Rani et al., 2000). Another feature-based determinant of trust is the level of automation, that is, whether the system is fixed, adjustable, or adaptive. It turns out that trust is higher when the system can be adjusted (Dietvorst et al., 2018; Hancock & Chignell, 1987), and consumers prefer automation that can learn, recognize, and respond to personality differences (Skjerve & Skraaning, 2004). Transparency is another influential feature of systems that affects trust. Numerous studies have shown that knowledge of the inner workings or logic of a system positively impacts trust (Dadashi et al., 2013; Dzindolet et al., 2003; Mercado et al., 2016).

The capabilities of a system that most strongly affect trust are the system’s consistency and reliability over time (McLeod et al., 2005). A system’s predictability and dependability engender trust and enable users to continue trusting the system over time (Cahour & Forzy, 2009; Muir, 1987). Users expect near-perfect performance from automation (Dzindolet et al., 2001), with system errors causing a rapid decline in trust (Dzindolet et al., 2001; Lee & Moray, 1994; Wiegmann et al., 2001). This decline in response to an error is stronger for algorithms than humans and is referred to as *algorithm aversion* (Dietvorst et al., 2015). The effect of automation failures on trust depends on the type and timing of errors, with greater negative impacts from errors on tasks that are easily performed by humans (Madhavan et al., 2006) and errors that occur early in interactions (Sanchez, 2006). The quality of information and service that automation provides further fosters satisfaction with the system, which is positively related to trust development (Donmez et al., 2006; Wang et al., 2011).

Second, user characteristics, such as self-confidence (Lee & See, 2004) and expertise (Fan et al., 2008), have been shown to significantly affect users’ level of trust in automation. Nonetheless, these enduring traits are not the primary focus of this study.

Third, external environmental factors impact users’ trust in automation. For example, task difficulty plays a role, with trust diminishing more when a system fails at simple tasks rather than complex ones, despite overall system performance surpassing human capabilities (Dzindolet et al., 2003; Madhavan et al., 2006). Additionally, task-related risk affects users’ trust in the system, although results have been ambiguous. While some researchers have found that users tend to reduce their reliance on automation as compared to humans when greater risk is involved (Ezer et al., 2008; Rajaonah et al., 2008), others have found the opposite effect (Lyons & Stokes, 2012).

Common to most of the above research on the influence of human, system, and environmental factors on users’ trust in systems is a view of trust as a steady-state variable rather than a dynamic attitude that evolves along with the interaction (Dzindolet et al., 2003). Indeed, only a few researchers have considered the dynamic nature of trust (Desai et al., 2013; J. D. Lee & Moray, 1994; J. Lee & Moray, 1992; Manzey et al., 2012; J. X. Yang et al., 2021; X. J. Yang et al., 2016, 2017). One of our objectives in the present study is to examine how trust evolves as a user undergoes repeated interactions with a system after the system has erred. In this way, we seek to gain a better understanding of how trust in systems may be rebuilt over long-term interactions.

Another consideration largely missing from prior research on trust in systems is what effect may arise from the choice of terminology used to describe the system. Studies in related fields have demonstrated that the choice of terminology can influence user behavior (Abraham et al., 2017). In the context of this study, terms like “smart systems,” “algorithms,” “artificial intelligence,” and “automated systems” are often used interchangeably to describe a computational formula that interacts autonomously with humans by processing inputs and generating outputs based on statistical models or decision rules. Most commonly, researchers have employed the terminology of traditional rule-based systems (e.g., “algorithms,” “automated systems,” and “decision support systems”). These researchers have found that users perceive problems arising from such systems to be more permanent than problems due to human error, as the former type of problem is perceived to result from standardization (Diaz & Ruiz, 2002). That is, traditional automated agents are thought to be invariant, whereas human agents are viewed as adaptable (Dijkstra et al., 1998; Höddinghaus et al., 2021). Automated systems are thus perceived to be incapable of learning (Dawes, 1979), incapable of considering individual targets (Grove & Meehl, 1996; Hamilton et al., 2021), and incapable of incorporating qualitative data (Grove & Meehl, 1996), but highly efficient (Galiere, 2020).

However, Langer et al. (2021) have shown that terminological differences used to refer to systems may directly affect users’ expectations, evaluations, and perceptions of a system. Specifically, referring to a system as “AI” seems to lead users to perceive the system as more complex in comparison to “decision support systems,” “automated systems,” “algorithms,” “computers,” or “technical systems” (Langer et al., 2021). A comparison of two studies illustrates this point: Lee (2018) and Marcinkowski et al. (2020) both had the same research objective, namely, to analyze the use of a system that automatically evaluates applicant information and recommends rejection or approval of applicants. Lee (2018) referred to the system as an “algorithm” and found that people preferred a human manager over the algorithm. Marcinkowski et al. (2020), on the other hand, framed the system as “AI” and found that users prefer the AI system over the human in hiring. Given Langer et al.’s findings, it is quite possible that the differing terminology played a substantial role in determining the disparate outcomes of these two studies.

In the present study, building on the research of Langer et al. (2021), we aim to offer additional empirical support for the hypothesis that what a system is called (i.e., the terminology used to refer to it) directly affects users’ level of trust in that system. We will also suggest ways in which previously established findings may need to be reevaluated considering the increasing popularity of terminology such as “artificial intelligence” (or “AI”).

### Attribution Theory and System Stability

To understand the dynamic nature of trust formation following a system failure, we draw on Weiner’s well-established causal attribution theory (1985), which is concerned with the cognitive processes by which people draw conclusions (“attributions”) as to the cause of a behavior (“causal attributions”). The theory has provided a useful framework for examining consumers’ behavioral response to service failures, including the impact of a failure on their level of trust and the rebuilding of that trust (Tomlinson and Mayer 2016) as well as the impact on customer satisfaction (Folkes et al., 1987; Hess et al., 2003).

In Weiner’s (1985) model, following a negative outcome, a trustor determines the outcome’s cause, evaluating that cause along the two attributional dimensions *controllability* and *stability*. The outcome of this evaluation determines the person’s behavioral response. Controllability indicates who or what is responsible for the outcome (Tsiros et al., 2004), whereas stability indicates the extent to which an outcome is likely to happen again. If the failure is due to a stable cause, this leads one to expect the same error in future situations with similar circumstances (i.e., negative outcomes recur under similar circumstances). Contrarily, an outcome due to a temporary cause fosters the expectation that future situations might lead to other outcomes (Weiner, 1985). If the event is perceived to be caused by temporary or fluctuating circumstances, the effect on the consumer’s reaction will be reduced (Hess et al., 2003; Lewicki et al., 1996).

Weiner (2000) concluded that attributions along the two dimensions of controllability and stability drive consumer reactions towards service errors. Although Weiner’s model was designed to study human–human interactions, the model puts little constraint on whom exactly is observed, and the theory is applicable in describing outcomes of any type of agent (van der Woerdt & Haselager, 2019). Thus, researchers have used Weiner’s attribution model to explain human-technology interactions. Findings show that users make stronger attributions of stability to technology-related causes than to human-related causes, resulting in stronger trust reduction in the former case (Belanche et al., 2020; Dijkstra et al., 1998). Hence, following a poor outcome of technology, users expect the system to be more likely to fail again, as the system cannot learn from its mistakes (Dawes, 1979), resulting in a negative effect on customers’ reactions. When the failure is due to a human, users expect the performance to be less stable, minimizing the negative effect on trust (Belanche et al., 2020).

Other scholars have argued along similar lines that users are reluctant to rely on systems because they believe that such systems will not fully consider the users’ unique individual circumstances (Longoni et al., 2019) and are unable to incorporate qualitative data (Grove & Meehl, 1996). Trust in systems is also lower because responsibility and blame cannot be shifted to a system and users have the implicit assumption that a system’s prediction should be perfect as the future is highly predictable, which is often not the case (Dawes, 1979). Further, users perceive systems to suffer from reductionism, meaning users think that certain qualitative information or contextualization is not being taken into account by the system which results in less accurate decisions (Newman et al., 2020). In contrast, unlike systems, humans are believed to be capable of improving through experience (Highhouse, 2008), detecting exceptions to the rule (Dietvorst et al., 2015), and providing explanations for their decisions (Armstrong, 1980).

Besides influencing trust, distinction in attributional controllability and stability also affects another user response, namely, satisfaction. Satisfaction following product failure is largely influenced by attributions of stability (Folkes, 1984). If a failure is attributed to stable factors, it leads to negative future expectations and lower satisfaction. However, if the failure is seen as having arisen from unstable causes, such as an isolated incident, it leads to positive expectations and higher satisfaction.

Modern systems are evolving beyond rigid rule-based structures, increasingly embracing self-learning capabilities. Such systems are predominantly labeled as Artificial Intelligence, or AI. Among ten terms most commonly used to refer to a system (e.g., “algorithms” and “decision support system”), “artificial intelligence” was found to be associated with the highest level of machine complexity (Langer et al., 2021), where the complexity of a system refers to the length of a concise description of a set of the entity’s regularities. Low complexity is ascribed to systems that are completely regular, whereas more complex systems are adaptive and are built to evolve strategies (Adami, 2002).

Drawing from attribution theory, then, the perceived high complexity of AI systems as compared to traditional rule-based algorithmic systems could potentially reduce stability ascription, which in turn could lead to a minimized effect on trust reduction as compared to algorithmic systems in case of failures, which are defined as a system’s performance falling below the user’s expectations (Hess et al., 2003). More specifically, the outcome variables of the present study are trust in the system, trust rebuilding, and overall satisfaction following the series of interactions, all variables which have previously been used as measures of behavioral response (Hess et al., 2003; Tomlinson and Mayer 2016). Hence, we suggest:

H1 Users’ behavioral response after system failure will be more negative for systems described as “algorithmic systems” than for those described as “AI systems”: (a) trust will be lower, (b) trust will be rebuilt more slowly, and (c) satisfaction will be lower.

H2 Attribution of stability mediates the effect of system type on behavioral response: stability attributions will be lower for systems described as “AI systems” than for “algorithmic systems.”

### Task Difficulty

Another relevant factor affecting trust after a system failure is the difficulty of the task in regard to which the failure occurred. Madhavan et al. (2006) found that automation failures on tasks that are perceived as easy have a greater negative impact on trust than failures on tasks that are perceived as difficult. This finding can be explained by attribution theory as follows. Weiner (1985) and Kelley (1973) suggest that consumers make controllability attributions either to internal or external causes. Effort and ability are seen as internal causes, whereas luck and task difficulty are perceived as external causes (Weiner, 1985). The importance of the internal properties of an agent might be discounted if there is high external justification for the task failure. That is, the presence of an external factor that impedes success (e.g., a difficult task) means that failure can be attributed, at least in part, to the task or environment rather than the actor, providing a basis for weaker attributions towards the actor in case of failure. Thus, failure at a more difficult task leads to a heightened external attribution (lower controllability) and suppresses internal attributions. If the failure is judged to be caused by the environment in this way and not by the properties of the actor, the cause is perceived to be less stable (Anderson, 1983; Kelley, 1973). Building on this, one way to increase trust in algorithmic systems and reduce the difference between trust in AI systems and algorithmic systems is to increase task difficulty. Hence, we suggest:

H3 Task difficulty mitigates the effect of system type on behavioral response.

H3a When task difficulty is high, controllability attributions will decrease, which subsequently leads to reduced stability attribution compared to scenarios with lower task difficulty.

H3b Task difficulty acts as a mitigating factor, causing the difference in behavioral responses between algorithmic and AI systems to be less pronounced in more difficult tasks. This is attributed to the dampening effect of task difficulty on the influence of system type on behavioral response.

## OVERVIEW OF STUDIES

Across three studies we examine the effect of different system types on consumer behavior following a system failure. In our studies, we use interactions prior to the failure to form user expectations. Study 1 shows how the two key dimensions of attribution theory, namely, controllability and stability, can explain differing reactions users have towards what they perceive to be AI versus algorithmic systems. We show that system type affects consumers’ reactions solely through the stability attribution. Stability attributions are further significantly predicted by controllability attributions, which are lower for more difficult tasks. Study 2 tests the theorized model in a real world setting and shows that the difference can be mitigated by changing causal attributions to the controllability dimension. In the domain of stocks, behavioral response differs for system types. In the more complex task of cryptocurrencies, the difference is eliminated. Hence, we show that errors at more difficult tasks (less controllability attribution) mitigate the difference in behavioral response between the system types. Study 3 demonstrates how changing causal attributions to the stability dimension by providing social account also eliminates the difference in user behavior. This research complied with the American Psychological Association Code of Ethics. Informed consent was obtained from each participant.

### Study 1

#### Overview

The aim of Study 1 was to analyze the effect of system type on the dynamic nature of trust, trust restoration, and satisfaction. Further, we measured users’ perceptions of the key dimensions of attribution theory, namely, stability and controllability, to provide an account of our findings. Prior research has shown that trust is lost faster and more persistently (Dietvorst et al., 2015) with technological agents as compared to human agents. However, the systems in focus in most of those studies were rule-based systems. Drawing from attribution theory, these systems are ascribed high stability, leading to a strong and persistent decline in trust after a failure. The increasing predominance of self-learning AI systems in place of rule-based algorithmic systems may be expected to lead to consumer reactions of trust more similar to those observed after the failure of human agents.

Study 1 was an online study in which participants were asked to use real data to forecast students’ GPA scores. In every round, participants received information on a student and the forecast of an expert system on the respective student’s GPA score (Online Appendix A). Half of the participants were told that they received the forecast of an algorithmic system, the other half that they received the forecast of an AI system. In point of fact, the forecasts received by the two groups were identical (see below for details of the participant selection process and overall methodology). In consideration of the dynamic nature of trust establishment, loss, and rebuilding, we measured three aspects of participants’ behavioral response: trust in the system measured in every round (both before and after the error occurred), the pace at which trust was subsequently regained, and the level of overall satisfaction with the system.

Analyzing the results, we first conducted an independent t-test with trust after the error as the dependent variable and system type as the independent variable. Then, we considered trust rebuilding by conducting a mixed-model analysis with trust measured after every round as an independent variable. To explain our findings using attribution theory, we conducted a mediation model with satisfaction as the outcome variable, system type as the predictor (AI vs. algorithm), and the two attributional dimensions (stability and controllability) as mediators. We included perceived task difficulty as a second predictor.

#### Participants

We recruited 300 participants through Amazon’s Mechanical Turk (hereafter “MTurk”) and randomly assigned them to one of the two system type conditions. Participants received $1.50 for their participation, and the four participants with the best overall forecasting accuracy additionally each received a $5 bonus payment. For all studies, participants were excluded as outliers if (1) they failed to pass a simple attention check at the beginning of the study, (2) they were identified as speeders using Mahalanobis Distance (with threshold at .999 quantile of the χ2p) (Hair, 2009), or (3) their response quality was identified to be degraded using intraindividual response variability (IRV). Using IRV we calculated the “standard deviation of responses across a set of consecutive item responses for an individual” across all 12 trials (Dunn et al., 2018, p. 108). The IRV can detect degraded response quality, and research suggests removing respondents with unreasonably low IRV scores, as these may reflect straightlining responses (Dunn et al., 2018). Respondents with unreasonably high IRV scores were also removed, as these may reflect highly random responses (Marjanovic et al., 2015). On these grounds, 14 participants were excluded from the data analyses in Study 1 yielding a final sample size of 286 participants (M_age_ = 38 years, 60% male).

#### Procedure

The experimental task used here was adapted from Dietvorst et al. (2015). In our study, participants were told that their task was to forecast college students’ first-year GPA. Over the course of 12 rounds, all participants were given identical information on one real student per round and were asked to estimate the student’s first year GPA (see Online Appendix A). In each round, all participants received the same prediction for the respective student’s GPA, although this prediction was purported to be generated by one of two types of systems: Half of the participants were told that they received the prediction of an “algorithmic” system, the other half were told that they received the prediction of an “AI” system. The participants were introduced to the characteristics of the relevant type of system that had generated the prediction received (Table 1).

**TABLE 1:** Manipulation of the System Description Used in Study 1

Artificial Intelligence (AI) for GPA predictions AI models continuously learn from the accumulating data and outcomes through a self-learning process. Their learning capability is high and they make decisions by analyzing data and adapting
Algorithms for GPA predictions Algorithms make their predictions through a predefined rule-based decision process. Their learning capability is low and their decisions are based on the same rules repeatedly

To ensure that our manipulation was successful, we measured the participants’ understanding of the mechanical or analytical nature of the system that had generated their prediction (Belanche et al., 2020). Participants were asked to rate each system’s behavioral base on a 7-point semantic differential scale consisting of “low learning skills/high learning skills” and “based on repetition/based on analyzing and adapting” (*α* = .95).

Then, the actual forecasting task began. In order to increase engagement in the task, participants were told that the four participants with the highest forecasting accuracy would each receive a $5 bonus. After each round of forecasting, participants received feedback consisting of the respective student’s actual GPA, the participant’s prediction error, and the average prediction error across all previous predictions. To establish a baseline and familiarize each participant with the average performance of the respective system from which they received forecasts, the system’s prediction over the first five rounds (prior to the system’s failure) was kept close to perfect. Then in round six the system’s forecast provided to each participant was off by over 20%. After this error, from round seven onward, the system’s performance was again kept close to perfect. Participants’ trust in the system in every round was measured using a scale for trust in human-machine systems adapted from and validated by Jian et al. (2000). That is, after each round participants were asked *“How much confidence do you have in the system’s forecast?”* and rated their level of trust on a scale from 0% to 100%. This same item has been used by other researchers to analyze users’ responses to a system’s failure (Dietvorst et al., 2015).

Next, participants were asked questions about attributional dimensions, satisfaction, and demographics. The items used to measure attributions were adapted from previous studies (Higgins & LaPointe, 2012; Peterson et al., 1982). Controllability was measured by asking respondents to evaluate the system’s controllability over the major cause that led to the error on a scale from 1 (= a cause over which the system has no control) to 7 (= a cause over which the system has complete control). Stability was measured with two items involving the likelihood of a similar error occurring again on a scale from 1 (= not at all likely) to 7 (= very likely), *α* = .77. Satisfaction was measured with an item used by Vaccaro et al. (2018), where higher values indicated higher levels of satisfaction with the system (on a scale from 1 to 7). In addition, we measured perceived task difficulty by asking participants to rate the difficulty of predicting GPA scores on a scale from 1 to 7, where higher scores indicate higher perceived difficulty of the task.

#### Results and Discussion

First, we confirmed that our manipulation of system type was successful, and that the algorithmic system was perceived to be more rule-based and mechanical (*M* = 3.03) than the AI system (*M* = 6.49), *t* (183.6) = 16.62, *p* < .001). To analyze initial levels of trust after the error, we ran an independent t-test with trust in the system after the error as a dependent variable and system type (AI vs. algorithm) as an independent variable. Results showed a significant effect for system type (*F* (1,284) = 3.85, *p* < .001), whereby trust in what participants thought to be an AI system was significantly higher (*M* = 67.14) after the failure than was trust in what was thought to be an algorithmic system (*M* = 57.11).

**Figure 1.**
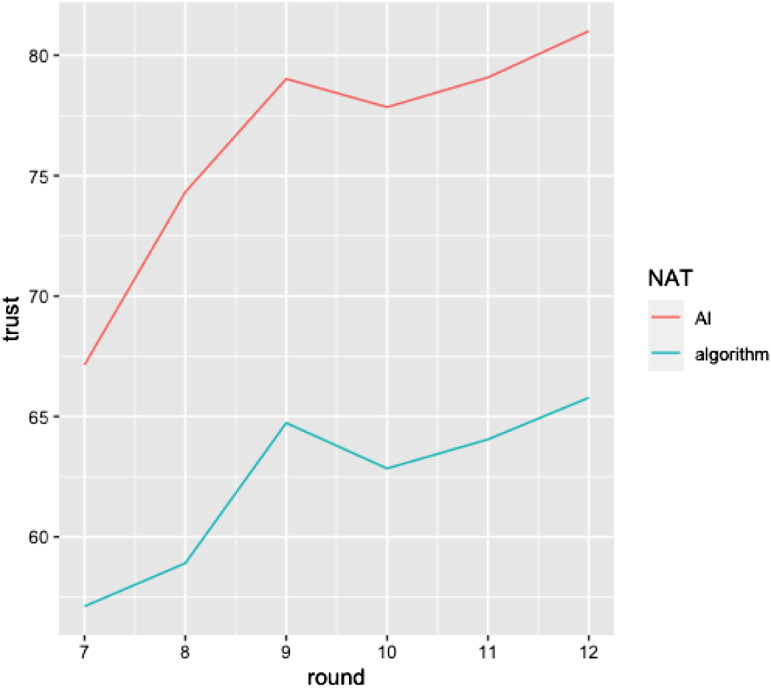
Trust regaining process after the occurrence of a system failure.

Next, we analyzed the effect of system type on trust rebuilding. To analyze how trust is rebuilt after seeing the system err, we ran a mixed-effects model of the six rounds that followed the mistake. Our outcome variable was trust in the system’s forecast as measured after each round. We added random intercepts for participants, allowing intercepts to vary across participants. The results reveal a nonsignificant main effect of type of system on trust (*ß* = −7.22, *SE* = 3.73, *t* (1719) = −1.94, *p* = .054), a significant main effect for number of rounds (*ß* = 2.36, *SE* = .24, *t* (1719) = .77, *p* < .001), and a significant interaction effect of system type and number of rounds (*ß* = −.73, *SE* = .33, *t* (1719) = −2.19, *p* = .029) (see Figure 1). Hence, overall, trust was rebuilt more rapidly for the AI system in the early rounds compared to the algorithmic system, highlighting the accelerated trust recovery in AI systems.

Finally, in order to explain the underlying process we conducted a mediation analysis, which included as mediators (Hayes, 2017) the causal attribution dimensions of Weiner’s attribution theory responsible for explaining postconsumption reaction following a service failure (Weiner, 2000). Perceived task difficulty was also included as a predictor. The effect of system type on satisfaction with the system was tested through the two attributional dimensions controllability and stability as mediators. As Preacher et al. recommend (Preacher et al., 2007), we used bootstrapping to test multiple mediator models, and we report bootstrap estimates derived from 10,000 bootstrap samples along with bias-corrected 95% confidence intervals.

**Figure 2.**
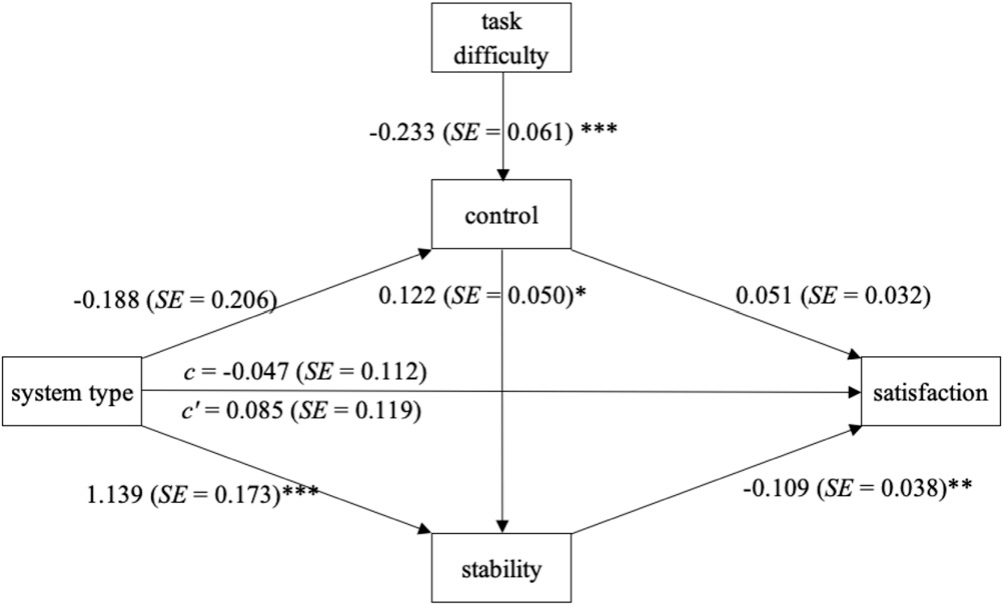
Attribution of stability mediates the effect of system type on satisfaction (*p < .05, **p < .01, and ***p < .001).

Figure 2 shows the paths diagram of the parallel mediation analysis. System type was coded as 0 = AI and 1 = algorithm. Task difficulty predicts controllability attributions (*ß* = −.233, *SE* = .061, *p* < .001), meaning more difficult tasks were perceived by the study participants to be less controllable. System type predicts stability attributions (*ß* = 1.139, *SE* = .173, *p* < .001) but not attributions of controllability (*ß* = −.188, *SE* = .206, *p* = .364). However, attributions of control predict attributions of stability (*ß* = .122, *SE* = .050, *p* = .015). Finally, only attributions of stability significantly predict satisfaction (*ß* = −.109, *SE* = .038, *p* = .005). The bias-corrected confidence intervals for the partially standardized indirect effects confirm this pattern: The only significant indirect effect of system type on satisfaction is through the mediator attributional stability, 95% CI [−.236, −.039]. The only indirect effect of task difficulty on satisfaction is the effect of task difficulty on controllability, which in turn affects stability, and ultimately impacts satisfaction, with a 95% CI [.001, .014]. All other indirect effects of task difficulty and system type on satisfaction are insignificant.

Thus, as expected, there is a significant indirect effect of system type on satisfaction rating, an effect mediated by stability attribution. Purported algorithmic systems are attributed higher stability, which in turn decreases satisfaction ratings. In addition, tasks that are perceived to be more difficult result in lower attribution of control, which leads to lower stability attributions, and in turn to higher satisfaction after seeing a system err.

Taking the results together, we demonstrate that an error by what users think to be an algorithmic system has a more negative impact on users’ attitudes and behavioral intentions towards the system than the same error has by what users perceive to be an AI system. We show that this phenomenon can be explained through attribution theory, whereby reduced stability attribution towards novel, self-learning systems mitigates the negative impact of the system’s error on behavioral intentions towards the systems. Our results show that system type does not affect the attributional dimension control but is mediated by stability attributions. Based on these results, we suggest that more difficult tasks will lead to decreased stability attributions, which mitigate the negative effect of a system’s error on behavioral intentions. These findings are consistent with prior findings on behavioral reactions towards human agents versus algorithms (Dietvorst et al., 2015) and suggest the same underlying functioning: Decreased stability ascription towards humans as compared to algorithms results in the same effect as decreased stability ascriptions towards AI systems.

### Study 2

#### Overview

Study 2 tests our proposed framework in another domain (finance). With this study we wanted to confirm whether increasing task difficulty changes the causal attribution of the first attributional dimension, controllability, and mitigates the differing effect of system type on behavioral responses caused by the second attributional dimension, stability. We used a 2 × 2 between-subjects design, which included the same manipulation of system type (algorithmic system vs. AI system) as Study 1. In addition, task difficulty was manipulated by using price forecasts in the domain of stocks versus the domain of cryptocurrencies (the latter forecasts being more difficult). Again, as dependent variables we included trust in the system (measured in every round before and after the error occurred), the pace at which trust is subsequently regained, and overall satisfaction with the system as central measures of behavioral response. When analyzing the results, we first conducted two independent t-tests, one for each domain, with trust after the error as the dependent variable and system type as the independent variable. Then, we analyzed trust rebuilding by conducting two mixed-model analyses, again one for each domain, with trust measured after every round as an independent variable. Finally, we conducted two independent t-tests for the domains with satisfaction as the dependent variable and system type as the independent variable.

#### Participants

700 were recruited to fill out an online survey via Amazon’s MTurk. Participants received $1.50 for their participation, and the participant who achieved the best overall forecasting accuracy additionally received a $20 bonus payment. Using the same rules as in study 1, 43 participants were removed from the sample resulting in a final sample size of 657 participants (M_age_ = 43 years, 59% male).

#### Procedure

The task procedures and measurements used for the study were adapted from prior research by Prahl and van Swol (2017) but using a financial task similar to the one used by Önkal et al. (2009). Participants were first provided with a short task description. Half of the participants were randomly assigned the task of forecasting weekly closing prices of stocks, the other half of cryptocurrencies. The game consisted of 12 rounds in total. In each round, participants received information on the performance history over the past 10 weeks of one stock or cryptocurrency. The 10-week closing prices and the final closing price in week 11 were based on real data obtained from the publicly available source MarketWatch. In this way, we kept the scenario as close to reality as possible, however leading to a difference between the stock and cryptocurrency data. The particular stock’s/currency’s name was not disclosed, but all of them are publicly traded. Based on the information provided, participants had to forecast the respective stock’s/cryptocurrency’s price at the regular market closing time. Half of the participants in each of the above subject pools were then provided with an estimate from what they were told was an algorithmic system, the other half from what was described as an AI system. Based on the system estimates provided, participants could reestimate the closing price and rate their trust in the system. To reduce potential biases, the system’s prediction was calculated so that the forecasting error in percentage was equal between the two system groups. Trust in the system was measured using the same item and scale from Jian et al. (2000) used in Study 1. Trust was measured in this way after each round and was the central dependent variable for the study. After each round, participants also received feedback on the actual price at closing time, the accuracy of their postadvice forecast, and their average forecast error across all previous forecasts.

To ensure that the manipulation of task difficulty would be successful, all participants received the following information following the task description:

The key differences between investing in stocks and investing in cryptocurrencies are:

The market for cryptocurrencies is generally a highly unstable market and prices move very aggressively. In comparison, the stock market is a much more stable market with less price fluctuations.

The exact description for the stock condition can be found in Online Appendix B. (If participants were ascribed to the cryptocurrency condition, the word “stock” was exchanged by “cryptocurrency.”).

Similarly, each participant was introduced to the characteristics of their respective system, either algorithmic or AI, the same as in Study 1. Also as in Study 1, the same scale from Belanche et al. (2020) was again used to ensure participants’ understanding of the mechanical or analytical nature of the system (*α* = .91). Next, participants were shown an example of the information that they would receive in the actual rounds to come and an explanation of the financial data (see Online Appendix C).

After this, the real trials began. Forecasts given over the first five rounds were kept close to perfect for both types of systems in order to establish a baseline and familiarize participants with the system and its average performance. The mean absolute percentage error in every trial is between .7% and 1.09%, which were comparable to the forecasting errors of a real AI model based on the same financial data of other stocks (Vijh et al., 2020). As in Study 1, the forecasts presented were identical across system groups, i.e., for that half of the participants being told that their system was algorithmic, and the other half being told that their advisor was AI. However, the absolute forecast numbers differed between cryptocurrency and stock groups, because the data was based on real data. Then, in round six the system’s forecast was off by almost 20%. After this failure, the systems performed again close to perfect for the remaining rounds.

After completing the 12 rounds, we measured perceived task difficulty using three adapted measures from Diacon (2004) (*α* = .73). As in Study 1, satisfaction was measured with an item from Vaccaro et al. (2018). The study ended by gathering responses to general demographic questions.

#### Results and Discussion

Results showed that our manipulation of system characteristics was successful, and that the system identified as algorithmic was perceived to be more mechanical and based on repetition (*M* = 3.34) than the AI system (*M* = 6.38), *t* (433.06) = 22.63, *p* < .001). We ran a 2 × 2 ANOVA with participants’ trust in the system after seeing it err (round 7) as the dependent variable and task difficulty (cryptocurrency vs. stock) and system type (AI vs. algorithm) as independent variables. Results showed a significant effect for system type (*F* (1,652) = 7.94, *p* = .005), with trust ratings for AI systems being higher (M = 67.07) than for algorithmic systems (M = 62.65). There was also a significant effect for task difficulty (*F* (1,652) = 4.94, *p* = .027), with trust ratings being higher for high task difficulty (M = 66.58) than for low task difficulty (M = 63.24). The interaction effect was not significant (*F* (1,652) = 2.34, *p* = .122).

We then ran the same ANOVA analysis but additionally controlled for trust ratings in the round prior to the mistake. The results confirmed the significant main effect of system type (*F* (1,651) = 17.82, *p* < .001) and task difficulty (*F* (1,651) = 11.09, *p* = .001), along with the insignificant interaction effect (*F* (1,651) = .937, *p* = .333). To test our hypothesis, we computed two independent t-tests, one for high task difficulty and one for low task difficulty. Results showed that for low task difficulty, trust was significantly higher for AI systems (*M* = 66.49) than for algorithmic systems (*M* = 59.44), *t* (312.63) = 3.08, *p* = .002. For high task difficulty, trust did not significantly differ between the AI (*M* = 67.66) and the algorithmic systems (*M* = 65.47), *t* (312.21) = 1.01, *p* = .311.

To analyze how trust is rebuilt after consumers have seen the system err, we ran two mixed-effects models of the six rounds that followed the mistake: one for high task difficulty and one low task difficulty. We ran two models because the underlying data presented to our participants differed between the cryptocurrency and stock groups. Therefore, directly comparing the two groups could lead to erroneously disregarding other underlying factors, such as the effect of different absolute values presented and the effect of greater variations between the closing price and the forecasts. Our outcome variable was trust in the system’s forecast as measured in each round. We added random intercepts for participants, which allowed intercepts to vary across participants.

For low task difficulty, the results showed a nonsignificant main effect of type of system on trust (*ß* = −4.59, *SE* = 3.01, *t* (1914) = −1.52, *p* = .129), a significant effect of number of rounds on trust (*ß* = −1.23, *SE* = .16, *t* (1914) = 7.52, *p* < .001), and a significant interaction effect of number of rounds and type of system on trust (*ß* = −.53, *SE* = .24, *t* (1914) = −2.21, *p* = .027). For both systems, the growing number of rounds increased trust in the system. However, trust per round increased less strongly for the algorithmic system than for the AI system. Hence, following a system error, trust in what is thought to be an AI system is regained more quickly than trust in what is thought to be an algorithmic system.

The same analysis for high task difficulty showed slightly different results: a nonsignificant main effect of type of system on trust (*ß* = −.60, *SE* = 2.87, *t* (2022) = −.21, *p* = .834), a significant effect of number of rounds on trust (*ß* = 1.05, *SE* = .16, *t* (2022) = 6.74, *p* < .001), but an insignificant interaction effect of number of rounds and type of system advisor on trust (*ß* = −.32, *SE* = .22, *t* (2022) = −1.43, *p* = .154). Again, the number of rounds increases trust in the system. However, unlike tasks with low difficulty, there is no difference in trust towards algorithmic and AI systems when a task with high difficulty is involved.

We also ran a 2 × 2 ANOVA with task difficulty and system type as independent variables and satisfaction as the dependent variable. Results showed a significant main effect for system type (*F* (1,651) = 7.14, *p* = .007), a significant interaction of system type and task difficulty (*F* (1,651) = 4.06, *p* = .044), and an insignificant main effect of task difficulty (*F* (1,651) = .08, *p* = .780). To further analyze the interaction effect, we computed an independent t-test for each condition of task difficulty. For low task difficulty, satisfaction was significantly higher with the AI system (*M* = 6.05) than with the algorithmic system (*M* = 5.61), *t* (278.02) = 3.390, *p* = .001. For high task difficulty, satisfaction did not differ with the AI system (*M* = 5.89) and the algorithmic system, (*M* = 5.83), *t* (332.00) = .501, *p* = .617.

In Study 1, we showed how the effect of system type can be explained in terms of attribution theory and suggested that more difficult tasks should mitigate the effect of system type on behavioral response. In Study 2, we have shown that increasing task difficulty indeed mitigates the negative effect on behavioral response of errors by what users think to be algorithmic systems. Study 2 aimed to examine the influence of modifying the first attribution dimension, assumed to be controllability, on behavioral response to different system types, without measuring controllability or stability. The combination of Studies 1 and 2 might imply that the results observed could be attributed to controllability.

### Study 3

#### Overview

Study 3 aims to show how altering causal attributions to the system, the second attributional domain, affects the user’s behavioral response.

Literature has identified four immediate actions that may improve the restoration of trust after it is lost (Lewicki et al., 1996). These are acknowledging the violation, determining the cause of the violation and admitting fault, admitting the act was destructive, and accepting responsibility for the consequences. Likewise, research by Dzindolet et al. (2003) has shown that offering a rationale for a machine’s failure may improve trust restoration.

According to attribution theory, initial judgments of a source’s (in this case, the system’s) trustworthiness after a negative outcome are not always final but can potentially be modified by taking into account additional information (Weiner, 1985). One type of reparative effort by the trustee that has been shown to be effective is the provision of *social accounts* (Scott & Lyman, 1968; Tomlinson et al., 2004). Social accounts are statements made to explain unanticipated behavior and to bridge the gap between expectations and outcomes (Scott & Lyman, 1968). They are a way to explain actions and decisions through justifications and excuses and play a crucial role in conflict management as a means of influencing the perception of the other party regarding an incident or situation. Communication and “talk” play a crucial role in the use of social accounts, and the ease of accessibility is what sets it apart from other reparative efforts such as forcing, smoothing, and collaboration. The one-way communication form of social accounts makes it more readily available and accessible, providing an effective means of explanation. Thus, providing information asserting that the cause has a more external locus, is uncontrollable, and/or is due to an unstable cause may positively affect trust repair (Weiner et al., 1987, 1991). Hence, especially rule-based systems, to which high stability is attributed, should strongly benefit from providing a rationale for the failure.

As Study 1 shows and Study 2 suggests, trust repair is higher for what users perceive to be AI systems than for algorithmic systems due to the lower stability attribution made towards AI systems. Reducing stability attribution towards algorithmic systems by the provision of social accounts should, therefore, more strongly affect trust repair for these systems. For these reasons, we hypothesize the following:

H4 The effect of system type on behavioral response is mitigated by providing social accounts for the mistake.

For Study 3, we used the same stock price forecasting task as in Study 2 (now without the cryptocurrency condition). We also used the same manipulation of system type, but for Study 3 we additionally manipulated social account by providing explanations to half of the participants of why the mistake occurred and no explanation to the other half of the participants. To analyze participants’ trust after the error, we ran an ANOVA with trust after the error as the dependent variable and system type and social account as independent variables. As with the other studies, to document the dynamic nature of trust we ran a mixed-model analysis with trust level after each round after the error as the dependent variable and system type and social account as the independent variables. Finally, we analyzed satisfaction ratings via two independent t-tests, one for the condition with social account and one for the condition without social account, with system type as the dependent variable.

#### Participants

Six hundred participants were recruited to fill out an online survey via Amazon’s MTurk. All participants received $1.50 for their participation, and the participant with the best overall forecasting accuracy additionally received a $20 bonus payment. Based on the same rules as in the two previous studies, 39 participants were removed from the sample. These eliminations resulted in a final sample size of 561 participants (M_age_ = 44 years, 56% male).

#### Procedure

Participants were randomly assigned to one of four conditions in a 2 × 2 between-subjects design (AI system vs. algorithmic system; social account vs. no social account). The manipulation of system type was identical to the one used in Study 2, and the overall procedure was the same as the one for Study 2 (in the stock condition), except that after the error occurred, half of the participants received a brief social account explaining the unanticipated behavior and bridging the gap between expectations and outcome. The exact information given to participants in the social account condition can be found in Online Appendix D. Satisfaction was measured using the same three items from Vaccaro et al. (Vaccaro et al., 2018) as before (*α* = .79).

#### Results and Discussion

We ran a 2 × 2 between-subjects ANOVA with social account (yes/no) and system type (AI/algorithm) as independent variables, and trust rating after the mistake as a dependent variable. The results reveal a significant main effect of system type (*F* (1,557) = 6.61, *p* = .010) and no significant main effect of social account (*F* (1,557) = .02, *p* = .889) or interaction effect (*F* (1,557) = .04, *p* = .851). Further analysis of the effect of system type shows that for purported AI systems, trust ratings were significantly higher (M = 64.68) than for purported algorithmic systems (M = 60.17), *t* (558.99) = 2.58, *p* = .010.

To rule out an effect of prior trust in the system, we ran the same model again, but now additionally controlling for the participants’ trust rating in the round before the mistake occurred. In this way, we could isolate the effect of the mistake from the overall effect of the system type. The results confirm the significant main effect of system type on trust rating (*F* (1,556) = 13.16, *p* < .001). This model confirms that after a mistake, users’ trust in what is believed to be an algorithmic system is significantly lower than their trust in what they believe to be AI system, meaning a mistake of an AI system impacts trust in the system less than a mistake of an algorithmic system.

To analyze whether social account has the expected effect on the regaining of trust, we ran a mixed-effects model. Our outcome variable was trust in the system’s forecast as measured in each round after the mistake. We added random intercepts for participants, thus allowing intercepts to vary across participants. Results show that the main effects for system type (*ß* = −2.84, *SE* = 3.30, *t* (3366) = −.86, *p* = .390) and social account (*ß* = −1.06, *SE* = 3.40, *t* (3366) = −.31, *p* = .755) are not significant. As with Study 2, the main effect of number of rounds is significant (*ß* = 1.24, *SE* = .18, *t* (3366) = 6.75, *p* < .001). Furthermore, there is a significant three-way interaction of system type, social account, and number of rounds (*ß* = .77, *SE* = .38, *t* (3366) = 2.01, *p* = .044). To analyze the interaction effect, we ran two separate models, one for the AI system and one for the algorithmic system. For the AI system, only the number of rounds significantly predicted trust (*ß* = 1.24, *SE* = .18, *t* (1662) = 6.88, *p* < .001), meaning that trust increased along with the increasing number of rounds. Social account had no significant effect on trust ratings (*ß* = −1.06, *SE* = 3.31, *t* (1662) = −.32, *p* = .749), and there was no significant interaction effect (*ß* = .06, *SE* = .27, *t* (1662) = .24, *p* = .808). For the algorithmic system, there was a significant main effect for number of rounds (*ß* = .90, *SE* = .19, *t* (1704) = 4.66, *p* < .001), no significant main effect for social account (*ß* = −4.38, *SE* = 3.42, *t* (1704) = −1.28, *p* = .202), and a significant interaction effect for number of rounds and social account (*ß* = .83, *SE* = .27, *t* (1704) = 3.05, *p* = .002).

We also computed two independent t-tests for satisfaction ratings, one for the group with social account and one for the group without social account. These tests showed that when no social account was provided, satisfaction was higher for the AI system (*M* = 5.92) than for the algorithmic system (*M* = 5.70), *t* (260.82) = 2.13, *p* = .034. When a social account was provided, satisfaction for the AI system (*M* = 5.82) and algorithmic system (*M* = 5.84) did not significantly differ, *t* (267.78) = −.23, *p* = .816.

**Figure 3.**
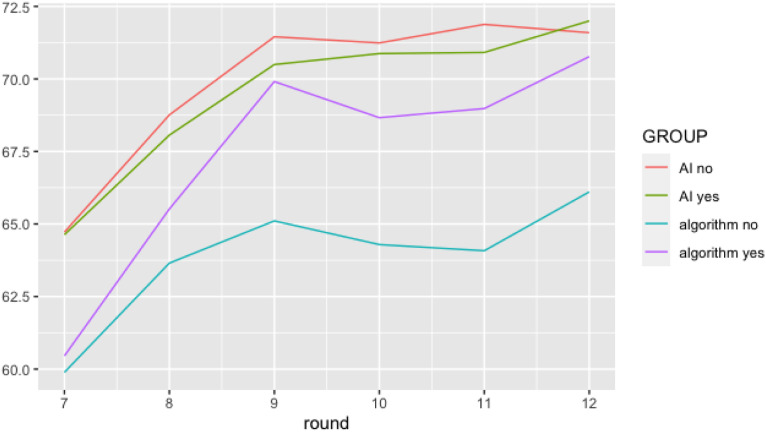
Trust regaining process after the occurrence of a system failure. Plotting level of trust over 7 consecutive rounds shows that social account (social account = yes) positively affects the regaining of trust in algorithmic systems (with high stability ascription) but not AI systems (with low stability ascription).

Based on these results, Figure 3 illustrates that for purported algorithmic systems, trust can be regained faster by providing a social account. However, for purported AI systems this is not the case, thus confirming H4.

## DISCUSSION

### Theoretical Contributions

Our research offers four notable contributions to the literature on trust in systems. First, we go beyond the traditional view of trust as a static variable by examining the dynamic nature of trust in recurring interactions. We focus on analyzing user behavioral responses to a system’s error, considering trust in system advisors as an evolving variable over time. Consequently, we investigate the levels of trust before and after a system’s predictive error, the sequential process of rebuilding trust, and consumer satisfaction throughout the entire interaction series.

Second, we demonstrate that the terminology and descriptions used for systems significantly influence consumer responses to system failures. In various contexts, such as media, policy making, or research, systems are referred to by different terms like “artificial intelligence” (AI), “algorithms,” or “decision support systems.” However, previous research indicates that the characterization of a system (e.g., “AI” vs. “algorithms”) impacts user expectations (Langer et al., 2021). Building on Dietvorst et al. (2015), we reveal that portraying a system as “AI” (a self-learning and more complex system) rather than “algorithmic” (a simpler rule-based system) results in more positive user responses after system errors.

Third, our research uncovers new insights into how system terminology influences causal attributions, which subsequently affects trust rebuilding and satisfaction. We also explore the impact of perceived system complexity variations on algorithm aversion. By contrasting AI systems (perceived as more complex) with algorithmic systems, we determine that user reactions to errors depend on the perceived system category. Mediation analysis further explains this effect using attribution theory (Kelley, 1973; Weiner et al., 1976). Stability ascription is higher for algorithmic systems than AI systems, and providing reasons for errors can change causal attributions. However, this adjustment is less effective for AI systems since stability ascription does not limit trust reestablishment. This shift in attribution also influences satisfaction ratings, with the difference between systems only being significant when no social account is offered.

Lastly, our fourth goal is to identify how the difference due to system classification can be mitigated. We showed that altering causal attributions towards lower controllability (by increasing task difficulty) as well as decreasing the system’s stability attribution (by offering social accounts) can mitigate the effect of system type on user behavior. As some systems are not self-learning and hence cannot be referred to as “AI,” we show how users’ tendency to respond relatively more negatively to algorithmic systems can be mitigated, either by changing controllability attributions (e.g., by noting the high complexity of the task involved) or causal attributions (e.g., by providing an explanation of the reasons for the system failure). The growing trend of firms falsely labeling their technology as “AI” when using rule-based systems, known as “AI washing” (Bini, 2018), erodes consumer and investor trust and hinders AI adoption (Vincent, 2019). This phenomenon can be compared to “autonowashing” (Dixon, 2020) in the driving automation domain, where misleading system naming impacts user expectations and trust. Studies have shown that the name of a driver assistance system influences user behavior (Abraham et al., 2017). Consequently, our findings offer guidance on how to lessen the negative reaction towards nonAI systems without resorting to “AI washing.”

These results have important implications for our understanding of algorithm aversion. Previous research results regarding trust in systems have been ambiguous (Castelo et al., 2019; Dietvorst et al., 2015, 2018; Logg et al., 2019; Marcinkowski et al., 2020). We suggest that part of the differences in findings may be due to the terminology used to describe the systems in question. A wide variety of terms have been used in previous studies to describe a system, including “artificial intelligence” (Marcinkowski et al., 2020), “algorithm” (Dietvorst et al., 2015, 2018; Lee, 2018), and “automated system” (Keel et al., 2018). Although these terms are similar, we have shown that the use of different terms may shape consumers’ perceptions of systems and consumers’ responses to these systems. Using the term “algorithmic system” may increase algorithm aversion, while using the term “artificial intelligence” may mitigate this same phenomenon. We thus highlight the importance of terminology, as this can unintentionally impact the robustness and replicability of research findings.

### Practical Contributions

Given the ubiquitous nature of automation and AI in various fields, our research findings bear significant practical implications across a range of applications. In media, policy making, research, customer care, and other domains, systems are variously referred to as “algorithms,” “decision systems,” “artificial intelligence,” and “automation,” amongst other terms. In our own present research, by comparing consumer behaviors in interactions with what are presented as “AI” versus “algorithmic” systems, we have shown that describing a system as “AI” triggers more beneficial responses. Following a service failure, customers interacting with what they believe to be an AI system are more willing to continue trusting the system, are more prone to rebuild any loss trust, and are more satisfied after the interaction than are customers who have been told they are interacting with an algorithmic system.

Emphasizing the practical relevance, it is crucial to recognize that such dynamics of trust and responses are not limited to mere consumer products but extend to domains where the stakes are high, such as medical diagnostics, financial advisory, and autonomous transportation. In healthcare, for instance, designating decision support systems as AI could foster higher levels of trust among patients and medical staff, especially following errors and inaccuracies, thus facilitating the adoption and efficient utilization of such systems in critical care settings. Likewise, in the financial sector, designating algorithmic trading systems as AI can potentially lead to increased investor confidence and resilience in the face of market volatility. Furthermore, in the realm of autonomous vehicles, clear and strategically chosen terminologies could help in establishing public trust which is vital for the broad acceptance of autonomous technologies.

However, it is important to acknowledge that not all systems are self-learning and can be labelled “AI” systems. This fact, together with the general perception of “AI” as desirable, has led to the practice of so-called “AI washing”: the mislabeling of technology to suggest that it delivers AI when in fact it does not. AI washing is harmful to the adoption of AI, as it undermines trust, acceptance, and development of the technology (Bini, 2018; Moore, 2017; Vincent, 2019). When a system’s characteristics do not allow for it to be called “AI,” our research suggests that it would be beneficial to provide users with an explanation of why the system’s error occurred. Similarly, our findings indicate that accounting for errors in terms of greater task complexity will likewise reduce consumer’s potentially negative responses to errors made by a less complex, algorithmic system.

In summary, our research suggests that companies should be strategically sensitive to the choice of terminology used to refer to advisor systems, as this choice may have a direct impact on consumer behavior toward the system and on the level of consumer satisfaction with the same. When transparency dictates that more desirable descriptors are unavailable for use, our research suggests that there are concrete ways to substantially mitigate the disadvantages this might otherwise entail.

### Limitations and Future Research

The present research raises several additional issues for researchers to consider. First, in the above studies we not only presented the terms “AI” and “algorithmic” as labels for the systems with which the participants interacted, but we also provided brief descriptions of the characteristics/capabilities of these systems. Thus, it may be that the participants were responding not simply to the use of the terms “AI” or “algorithmic,” per se, but instead to these participants’ understanding of the nature and capacities of the systems based on *the definitions/descriptions* of the systems that we provided. To determine how users respond to systems only in regard to the labels/terms themselves by which those systems are labelled (i.e., “AI” vs. “algorithmic”), we would have needed to *not* provide any additional definition/description of the systems. This is, of course, somewhat a catch-22 situation, as not providing a definition/description would introduce its own problem, namely, that without a definition/description to clarify the nature of the particular system, some or perhaps even many of the participants might not understand what the terms “AI” or (especially) “algorithmic” actually mean, making the results of such a study suspect for that reason. Thus, while we intentionally chose a more controlled design for the present studies, we suggest that the limitations imposed by this design pave the way for carefully designed future research using only the terms versus using the terms plus descriptions.

Second, our study designs did not include measures of trust in human agents. Adding a control group of participants who received forecasts by a human agent would have opened the possibility to directly test the degree of similarity between the response towards AI systems and human agents. We chose not to do so because prior research has already compared the behavioral responses to errors of systems versus humans (Dietvorst et al., 2015).

Third, the present research did not consider individual and contextual differences. However, individual differences in, for example, self-confidence (Lee & See, 2004) and expertise (Fan et al., 2008) have been shown to affect trust in systems. We encourage scholars to look further into the impact of these and similar factors.

Fourth, in our research, system failure was treated as a binary variable. In contrast to this research design, future research could manipulate system performance in more continuous ways (e.g., degree of forecasting accuracy and deviation from usual accuracy) and thereby possibly uncover other interesting differences between system types.

Lastly, it is crucial to exercise caution when relying on AI systems, as their showcased capabilities may not accurately reflect their real-world performance (Woods, 2016). As automation becomes more autonomous and takes on roles similar to team members, a relational approach to trust is needed, focusing on trusting rather than trust calibration (Chiou & Lee, 2023).

This approach considers not only the social influence of automation but also the social implications of people adjusting to and interacting with it. Measures of responsivity and the ability to resolve conflicting goals may be more relevant than reliability and reliance actions for future automation. By adopting a relational framework centered on situation, semiotics, sequence, and strategy, we can better understand the mechanisms and broader effects of trusting increasingly capable automation, fostering more resilient human-automation partnerships (Chiou & Lee, 2023).

## KEY POINTS

- Different terminology (e.g., “AI” vs. “algorithmic”) significantly influences consumer perceptions and responses, emphasizing the importance of strategic terminology use for trust and acceptance in industries using such systems.
- Describing a system as “AI” instead of “algorithmic” elicits more favorable behavioral responses during system errors, underscoring the impact of terminology on user behavior.
- When a system cannot be labeled as “AI”, providing users with an explanation of the error and highlighting task complexity can help mitigate negative responses and enhance system acceptance.

## Supplemental Material

Supplemental Material - How Terminology Affects Users’ Responses to System Failures Supplemental Material for How Terminology Affects Users’ Responses to System Failures by Cindy Candrian and Anne Scherer in Human Factors

## ORCID iDs

Cindy Candrian https://orcid.org/0000-0002-2177-8052

Anne Scherer https://orcid.org/0000-0003-4074-4859
