# Supplementary material for: How Terminology Affects Users’ Responses to System Failures
Source: Hum Factors. 2023 Sep 21;66(8):2082–103. doi: 10.1177/00187208231202572 (PMC11141081; doi:10.1177/00187208231202572)
Supplement: Supplemental Material - How Terminology Affects Users’ Responses to System Failures [file sj-pdf-1-hfs-10.1177_00187208231202572.pdf]

Appendix A

Participants received the following information on one real student in every round and were asked to predict the student's GPA:

HSGPA	SATV	SATM	Male	HU	SS	FirstGen	CollegeBound	GPA
3.83	680	770	1	3	9	1	1	?

HSGPA	High school GPA on a 0.0 to 4.0 scale
SATV	Verbal/critical reading SAT score
SATM	Math SAT score
Male	1= male, 0= female
HU	Number of credit hours earned in humanities courses in high school
SS	Number of credit hours earned in social science courses in high school
FirstGen	1= student is the first in her or his family to attend college, 0=otherwise
CollegeBound	1=attended a high school where >=50% students intended to go on to college, 0=otherwise
GPA	First-year college GPA on a 0.0 to 4.0 scale

Appendix B

Manipulation of system type used in Studies 2 and 3. The text used for the AI system was the following:

AI FOR PRICE PREDICTIONS

Artificial intelligence (AI) models are capable of identifying fundamental trends from data through a self-learning process. Their learning capability is high, and they make decisions by analyzing and adapting. Through intelligent data mining techniques, the models create a set of new variables using the financial dataset with Open, High, Low, Volume and Close of a particular company. For the prediction the model creates the following six internal variables:

1. Stock High minus Low price (H-L)

2. Stock Close minus Open price (O-C)

3. Stock price's seven days' moving average (7 DAYS MA)

4. Stock price's fourteen days' moving average (14 DAYS MA)

5. Stock price's twenty-one days' moving average (21 DAYS MA)

6. Stock price's standard deviation for the past seven days (7 DAYS STD DEV)

These new indicators play a crucial role in terms of training the models towards improved accuracy in predicting the next closing price of a particular company. The models learn continuously from the accumulating data and outcomes and have the ability to change, adapt and grow with time.

Manipulation of system type used in Studies 2 and 3. The text used for the algorithmic system was the following:

ALGORITHMS FOR PRICE PREDICTIONS

Algorithms are capable of identifying fundamental trends from data through a predefined rule-based decision process. Their learning capability is low, and their decisions are based on the same rules repeatedly. Through a step-by-step process the models create a set of new variables using the financial dataset with Open, High, Low, Volume and Close of a particular company. For the prediction the model creates the following six internal variables:

1. Stock High minus Low price (H-L)

2. Stock Close minus Open price (O-C)

3. Stock price's seven days' moving average (7 DAYS MA)

4. Stock price's fourteen days' moving average (14 DAYS MA)

5. Stock price's twenty-one days' moving average (21 DAYS MA)

6. Stock price's standard deviation for the past seven days (7 DAYS STD DEV)

These new indicators pay a crucial role in terms of enabling the system to come to a defined output based off a set of rules and predicting the next closing price of a particular company.

Appendix C

Explanation of the financial data in Study 3. The text in the stock condition was the following:

Task Information

In every round you will receive a **chart** showing the stock's closing price over the past 10 weeks and a **table** with the following weekly financial data:

1) Open: Open means the price at which a stock started trading when the opening bell rang
2) High: The high is the highest price at which a stock traded during a period
3) Low: The lowest is the lowest price at which a stock traded during a period
4) Volume: Volume is the total number of stocks traded over a period
5) Close: Close refers to the price of a stock when the stock exchange closed that week

The stocks' names will not be disclosed but all stocks are publicly traded US stocks.

Below you can find an example of the financial data you will receive in every round.

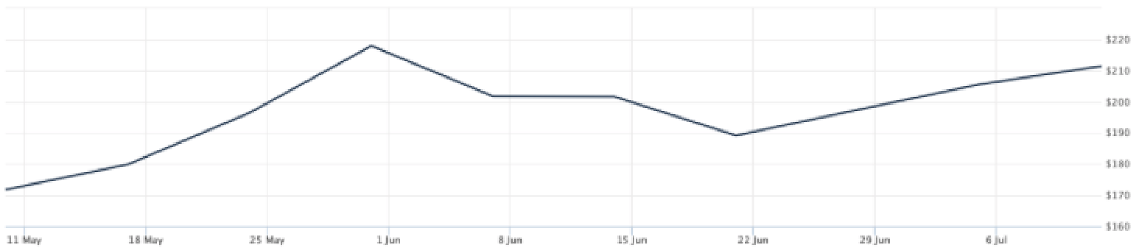

Date	Open	High	Low	Volume	Close
2020-05-11	182.54	184.85	165.36	16'251'200	**171.87**
2020-05-18	177.71	183.83	175.24	13'652'900	**179.93**
2020-05-25	185.63	211.31	185.22	22'863'900	**196.49**
2020-06-01	197.31	222.98	195.12	18'091'300	**217.92**
2020-06-08	220.02	222.26	194.02	17'134'100	**201.78**
2020-06-15	195.73	214.98	195.15	15'378'900	**201.63**
2020-06-22	200.99	207.51	188.51	20'012'600	**189.19**
2020-06-29	191.38	203.00	188.66	12'158'300	**197.40**
2020-07-06	201.34	207.79	195.00	16'785'800	**205.56**
2020-07-13	209.00	225.24	207.00	31'552'200	**211.41**
2020-07-20					**?**

Explanation of the financial data in Study 3. The text in the cryptocurrency condition was the following:

Task Information

In every round you will receive a **chart** showing the cryptocurrency's price at the stock market's closing time over the past 10 weeks and a **table** with the following weekly financial data:

1) Open: Open means the price at which the cryptocurrency started at the beginning of the period
2) High: The high is the highest price at which the cryptocurrency traded during a period
3) Low: The lowest is the lowest price at which the cryptocurrency traded during a period
4) Volume: Volume is the total number of the cryptocurrency traded over a period
5) Close: Close refers to the price of the cryptocurrency when the stock exchange closed that week

The cryptocurrencies names will not be disclosed but all cryptocurrencies are publicly traded.

Below you can find an example of the financial data you will receive in every round.

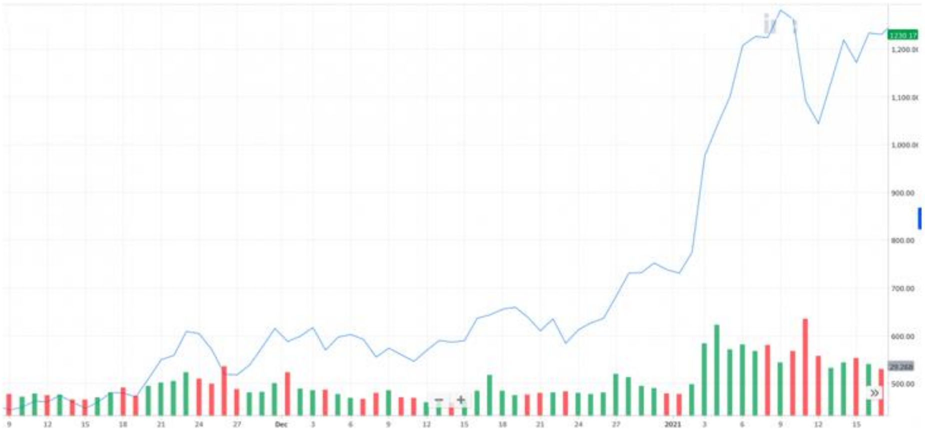

Date	Open	High	Low	Volume	Close
09.11.20	453.57	475.22	435.16	86559593873	**447.56**
16.11.20	447.56	579.37	445.50	117742517844	**558.07**
23.11.20	558.06	621.17	485.50	148365425751	**575.76**
30.11.20	575.76	635.16	563.11	121611808952	**601.91**
07.12.20	601.80	602.92	533.00	81351653045	**589.66**
14.12.20	589.78	673.83	577.12	100712316665	**638.29**
21.12.20	638.32	711.39	560.36	113119816165	**682.64**
28.12.20	683.21	1006.57	683.21	152747909037	**975.51**
04.01.21	977.06	1347.93	912.31	301834730092	**1262.25**
11.01.21	1261.62	1290.05	924.92	259298253002	**1230.17**
18.01.21					**?**

Appendix D

Social accounts text used for AI and algorithmic systems in Study 3:

AI system	Algorithmic system
The AI system noticed that its forecast error for the last prediction was larger than normal. This mistake will help the AI increase its accuracy for future predictions because it learns from its own mistakes.<br><br>**Why did the error occur?:** The main reason for the error was an overweighing of the stock price's 14 days' moving average (14 DAYS MA).	The algorithm noticed that its forecast error for the last prediction was larger than normal.<br><br>**Why did the error occur?:** The main reason for the error was an overweighing of the stock price's 14 days' moving average (14 DAYS MA).
